# Supplementary material for: The Constitutive Activity of Spinal 5-HT6 Receptors Contributes to Diabetic Neuropathic Pain in Rats
Source: Biomolecules. 2023 Feb 15;13(2):364. doi: 10.3390/biom13020364 (PMC9953062; doi:10.3390/biom13020364)
Supplement: Supplementary file 1 [file biomolecules-13-00364-s001.zip › biomolecules-2161400-supplementary.pdf]

Table S1. Detailed statistics for figures S1 and S2.

Figure	Analysis	Statistics (DFn, DFs)	P value
Figure S1a	1-way ANOVA	$F(2, 6)$= 4.226	P=0.0715
Figure S1b	1-way ANOVA	$F(2, 9)$= 6.788	P=0.0159
Figure S2a	2-way RM ANOVA	$F(14, 203)$= 7.586	P<0.0001
Figure S2b	1-way ANOVA	$F(2, 27)$= 13.93	P<0.0001
Figure S2c	2-way RM ANOVA	$F(10, 80)$= 5.018	P<0.0001
Figure S2d	1-way ANOVA	$F(2, 17)$= 8.8555	P=0.0023
Figure S2e, f	2-way RM ANOVA	$F(25, 190)$= 6.067	P<0.0001
Figure S2g	1- way ANOVA	$F(5, 38)$= 33.40	P<0.0001
Figure S3a, b	*2-way ANOVA*	$F(25, 200)$= 5.257	P<0.0001

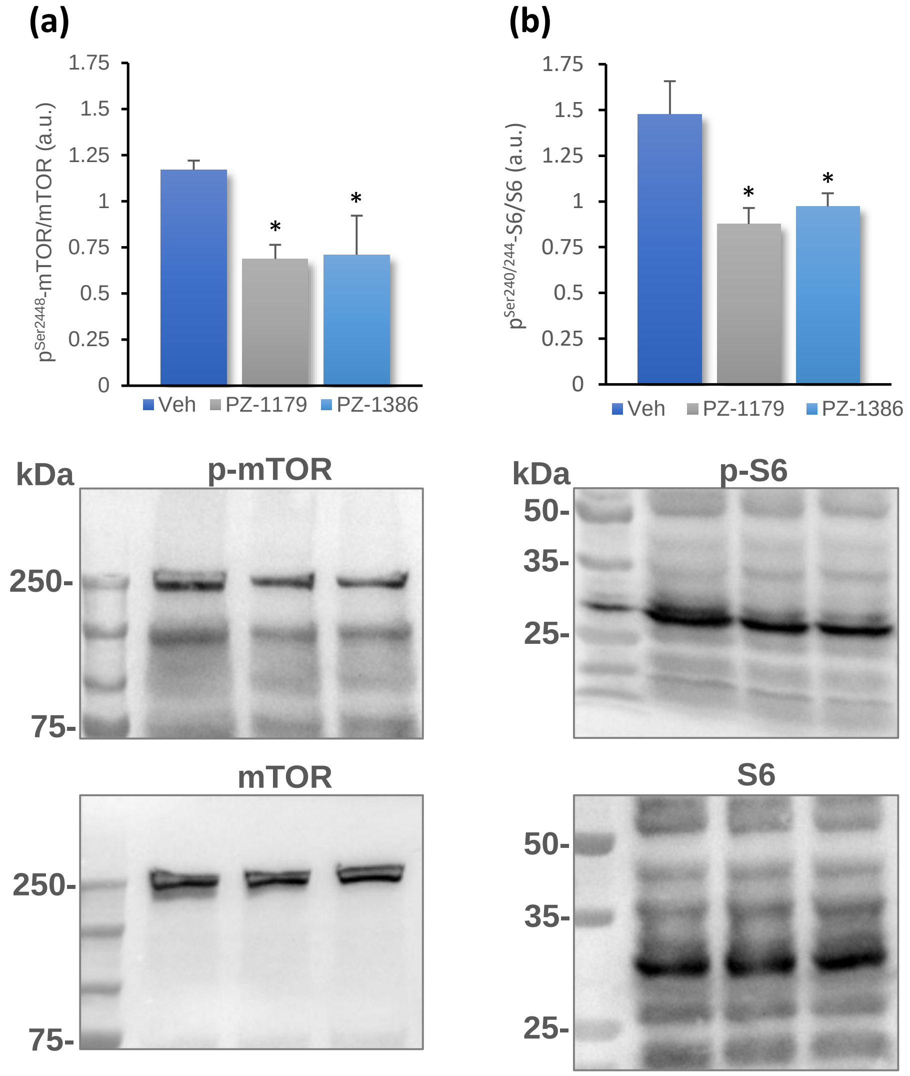

Figure S1. Inverse agonists reduce 5-HT_6 receptors constitutive activity at mTOR signaling. PZ-1179 and PZ-1386 reduces agonist-independent 5-HT_6 receptor-operated mTOR (**a**) and S6 (**b**) phosphorylation (at Ser^{2448} and $Ser^{240/244}$, respectively) in HEK-293 cells. HEK-293 cells expressing 5-HT_6 receptors were incubated with either vehicle (Veh), PZ-1179, or PZ-1386 (1 μM each) for 1h. *P<0.05 *vs*. vehicle, 1-way ANOVA.

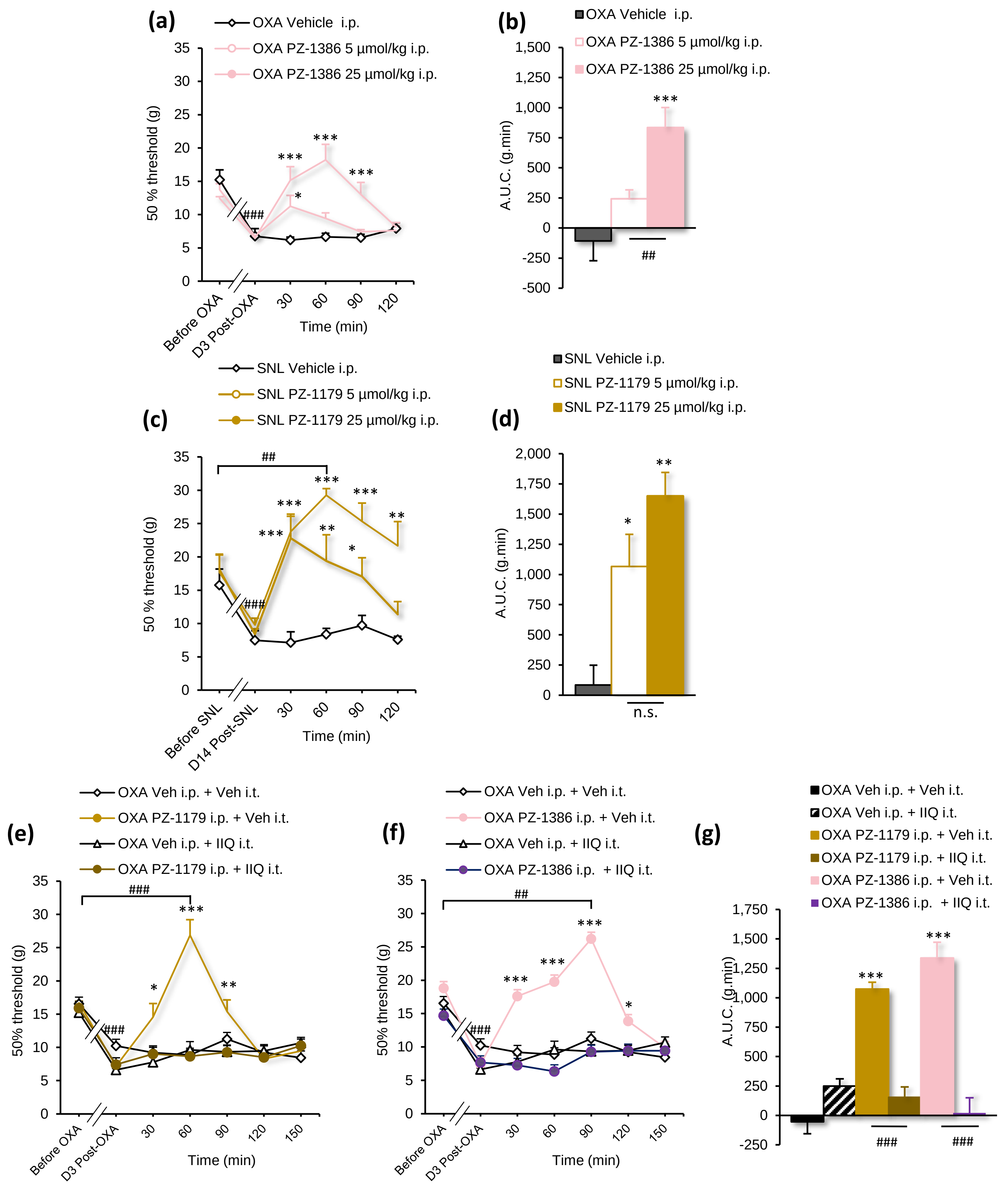

Figure S2. Effect of 5-HT$_6$ receptor inverse agonists on tactile hypersensitivity (von Frey hair test) in OXA and SNL rats. Intraperitoneal administration of PZ-1386 in OXA rats (**a**, **b**), PZ-1179 in SNL (**c**, **d**) and OXA (e) rats (5 and/or 25 µmol/kg each) but not vehicle (water for injections) suppresses mechanical hypersensitivity (n = 5-11/ group). 50% thresholds are expressed as grams (g). $^{###}$*P*<0.001 *vs.* values measured before OXA or SNL; **P*<0.05, ***P*<0.01, ****P*<0.001 *vs.* values measured before the drug/vehicle (water) injection (D3 Post-OXA or D14 Post-SNL), 2-way ANOVA; (**e**, **f**) Intrathecal administration of IIQ (2 nmol/rat) but not vehicle (water for injections, 10 µl/rat) suppressed the effect of (**e**) PZ-1179 (25 µmol/kg, i.p.) and (**f**) PZ-1386 (25 µmol/kg, i.p.) on paw withdrawal threshold induced by von Frey hair application (n = 5-8/group). $^{###}$*P*<0.001 *vs.* values measured before OXA; **P*<0.05, ***P*<0.01, ****P*<0.001 *vs.* values measured before drug/vehicle injection (D3 Post-OXA), 2-way ANOVA; (**b**, **d**, **g**) Area under the (A.U.C.) between 0-120 min of 50 % threshold variations in OXA and SNL rats calculated by the trapezoidal rule and expressed as g . min.. **P*<0.05, ***P*<0.01, ****P*<0.001, *vs.* OXA or SNL Vehicle group; $^{\#}$*P*<0.05 $^{###}$*P*<0.001 *vs.* values measured in corresponding group, 1-way ANOVA.

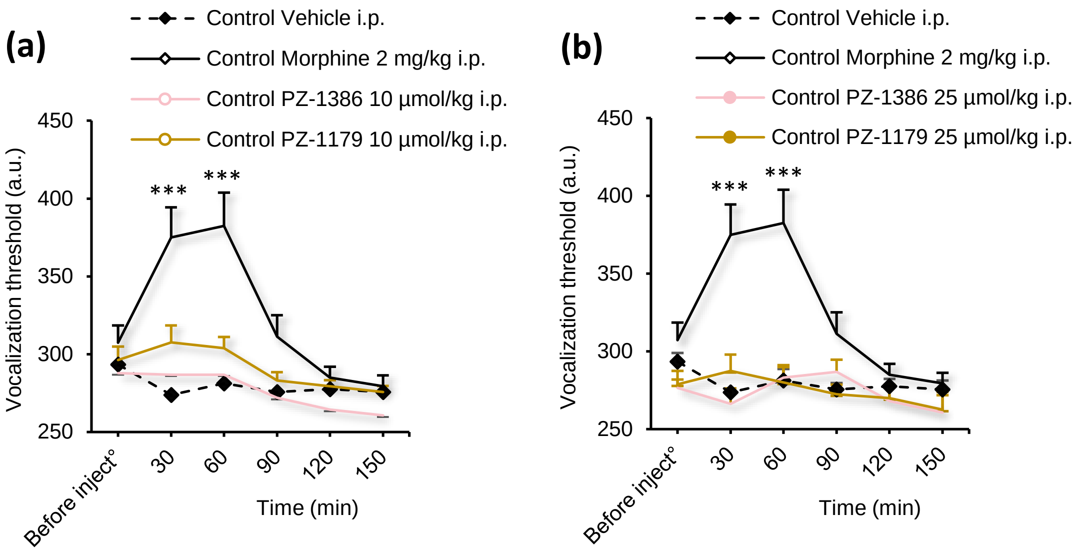

Figure S3. Effect of 5-HT$_6$ receptor inverse agonists on mechanical nociception in healthy control rats. Intraperitoneal administration of vehicle (water for injections), PZ-1386 or PZ-1179 at the dose of 10 µmol/kg (**a**) or 25 µmol/kg (**b**) fails to induce antinociception to paw pressure (n = 6-8/ group). Vocalization thresholds are expressed as arbitrary units (a.u.). ***P<0.001 *vs.* values measured before the drug/vehicle injection, 2-way ANOVA.
